# Crystal structure of 1-ethyl­spiro­[imid­az­olidine-4,1′-indane]-2,5-dione

**DOI:** 10.1107/S1600536814017097

**Published:** 2014-08-01

**Authors:** Wahraan Mohammed Hussein, Cynthia E. Theodore, S. B. Benaka Prasad, M. Madaiah, S. Naveen, N. K. Lokanath

**Affiliations:** a Department of Studies in Physics, University of Mysore, Manasagangotri, Mysore 570 006, India; b Department of Chemistry, School of Engineering and Technology, Jain University, Bangalore 562 112, India; c Department of Studies in Chemistry, University of Mysore, Manasagangotri, Mysore 570 006, India; d Institution of Excellence, University of Mysore, Manasagangotri, Mysore 570 006, India

**Keywords:** crystal structure, spiro compounds, hydantoin derivatives, {⋯HNCO}_2_ synthons, helical supra­molecular chain, C—H⋯O inter­actions

## Abstract

In the title compound, C_13_H_14_N_2_O_2_, the C_5_ ring has an envelope conformation with the C atom adjacent to the quaternary C being the flap. The five atoms comprising the imidazolidine-2,4-dione ring are almost planar (r.m.s. deviation = 0.004 Å). The dihedral angle between the five-membered rings is 89.66 (10)°. In the crystal, inversion-related mol­ecules are connected *via* {⋯HNCO}_2_ synthons. These are linked into a helical supra­molecular chain along [010] by C—H⋯O inter­actions.

## Related literature   

For background to the synthesis and biological activity of hydantoin derivatives, see: Manjunath *et al.* (2011 ▶, 2012 ▶). For conformational analysis, see: Cremer & Pople (1975 ▶).
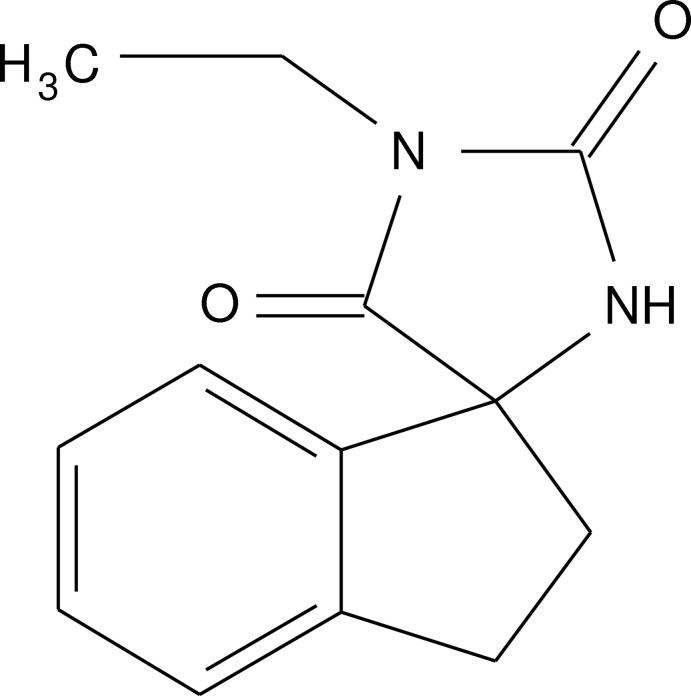


## Experimental   

### Crystal data  

C_13_H_14_N_2_O_2_
*M* *_r_* = 230.26
Monoclinic, 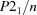

*a* = 13.7183 (10) Å
*b* = 6.2040 (5) Å
*c* = 15.1944 (11) Å
β = 112.865 (3)°
*V* = 1191.56 (16) Å^3^
*Z* = 4
Cu *K*α radiation
μ = 0.72 mm^−1^
*T* = 296 K
0.23 × 0.22 × 0.21 mm

### Data collection  

Bruker X8 Proteum diffractometer
Absorption correction: multi-scan (*SADABS*; Sheldrick, 1997 ▶) *T*_min_ = 0.867, *T*_max_ = 0.867
6730 measured reflections
1953 independent reflections
1742 reflections with *I* > 2σ(*I*)
*R*_int_ = 0.027

### Refinement  

*R*[*F* ^2^ > 2σ(*F* ^2^)] = 0.042
*wR*(*F* ^2^) = 0.120
*S* = 1.07
1953 reflections
156 parameters
H-atom parameters constrained
Δρ_max_ = 0.23 e Å^−3^
Δρ_min_ = −0.21 e Å^−3^

### 

Data collection: *APEX2* (Bruker, 2013 ▶); cell refinement: *SAINT* (Bruker, 2013 ▶); data reduction: *SAINT*; program(s) used to solve structure: *SHELXS97* (Sheldrick, 2008 ▶); program(s) used to refine structure: *SHELXL97* (Sheldrick, 2008 ▶); molecular graphics: *PLATON* (Spek, 2009 ▶); software used to prepare material for publication: *PLATON*.

## Supplementary Material

Crystal structure: contains datablock(s) global, I. DOI: 10.1107/S1600536814017097/tk5331sup1.cif

Structure factors: contains datablock(s) I. DOI: 10.1107/S1600536814017097/tk5331Isup2.hkl

Supporting information file. DOI: 10.1107/S1600536814017097/tk5331Isup3.cml

**Fig. 1.**
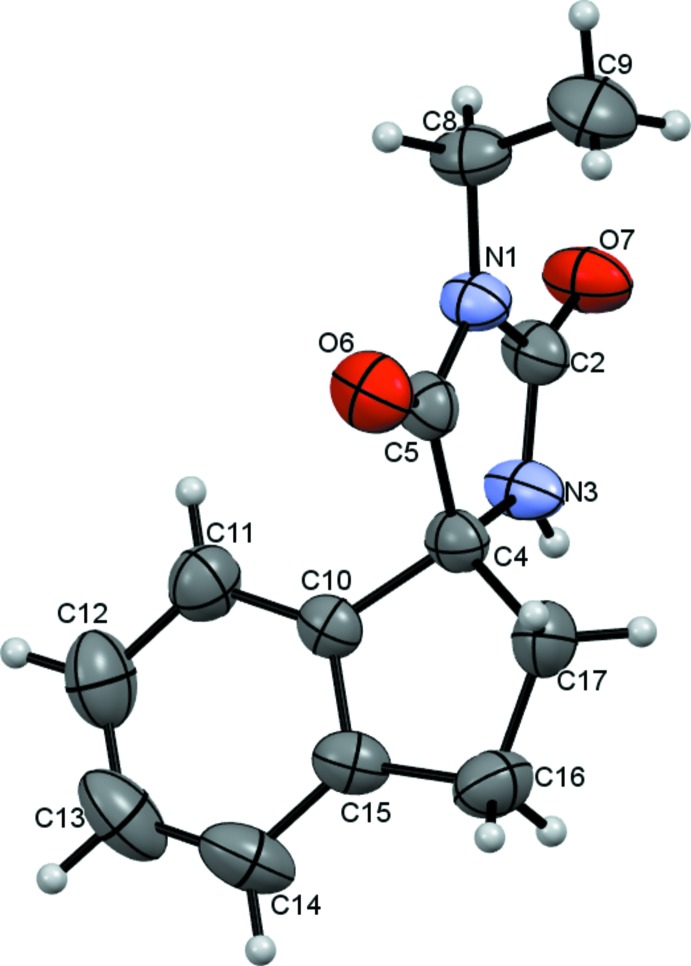
A view of the title molecule, with atom labelling. Displacement ellipsoids are drawn at the 50% probability level.

**Fig. 2.**
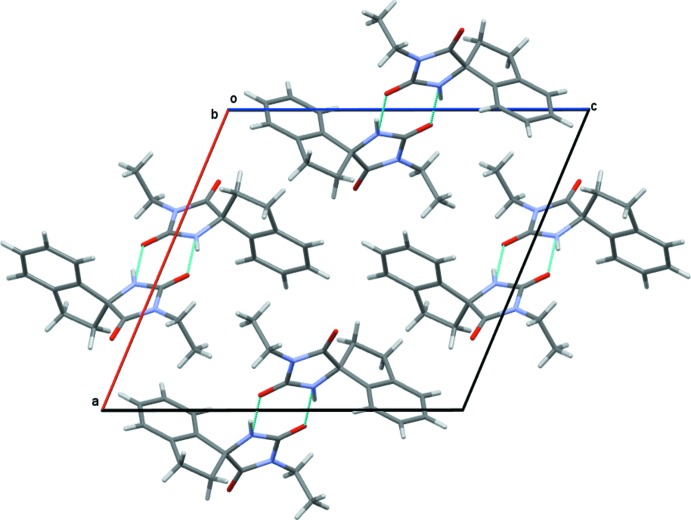
A view along the b axis of the crystal packing of the title compound.

. DOI: 10.1107/S1600536814017097/tk5331fig1.tif
A view of the title mol­ecule, with atom labelling. Displacement ellipsoids are drawn at the 50% probability level.

b . DOI: 10.1107/S1600536814017097/tk5331fig2.tif
A view along the *b* axis of the crystal packing of the title compound.

CCDC reference: 1015714

Additional supporting information:  crystallographic information; 3D view; checkCIF report

## Figures and Tables

**Table 1:** Hydrogen-bond geometry (Å, °)

*D*—H⋯*A*	*D*—H	H⋯*A*	*D*⋯*A*	*D*—H⋯*A*
N3—H4⋯O7^i^	0.86	2.05	2.886 (2)	163
C11—H7⋯O7^ii^	0.93	2.58	3.501 (2)	173
C17—H10⋯O6^iii^	0.97	2.56	3.392 (2)	143

Symmetry codes: (i) 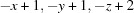; (ii) 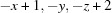; (iii) 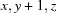.

## supplementary crystallographic information

### S1. Comment

The chemistry and properties of hydantoins and their derivatives have been investigated for more than 140 years. The hydantoin moiety which is present in various biologically active compounds is of immense pharmaceutical importance. There has been considerable interest in the synthesis and characterization of hydantoin derivatives as an important class of heterocyclic compounds. Hydantoin derivatives that display interesting activities against a broad range of biological targets have been identified. Activity of hydantoin derivatives depends on the nature of substitution of hydantoin rings. As a part of our ongoing research on hydantoins (Manjunath *et al.*, 2012), the synthesis, characterization and the structural work was undertaken on the title compound and herein we report its crystal structure.

The hydantoin ring in the structure is planar within experimental limits with a maximum deviation of 0.0036 (19) Å for C2 atom from the least squares plane of the hydantoin ring. The N—C bong lengths of N1—C2 = 1.394 (2) Å, N1—C5 = 1.367 (2) Å and N3—C2 = 1.337 (2) Å are comparable with the values reported earlier (Manjunath *et al.*, 2011; Manjunath *et al.*, 2012). The shortened bond length values can be attributed to the π-conjugation in the hydantoin ring.

The study of torsion angles, asymmetric parameters and least squares plane reveals that the five membered ring of the bicyclo octane moiety adopts envelope conformation with C4 atom deviating by 0.1121 (17) Å from the least-squares plane (Cremer & Pople, 1975). This is confirmed by the puckering amplitude *Q* = 0.2163 (19) Å. The hydantoin ring is in a equatorial position with the five membered ring which is evident by the dihedral angle value of 89.66 (10)°. The structure of the molecule is stabilized by the intermolecular hydrogen bonds of the type N—H···O and C—H···O (Table 1).

### S2. Experimental

To a solution of 2, 3-dihydrospiro-[imidazoline-4–1-indene]-2,5-dione (1.0 eq) in *N*,*N*-dimethylformamide was added anhydrous K_2_CO_3_ (3.0 eq) followed by stirring for 10 min. 1-Bromoethane (1–1.1eq) was then added. The reaction mixture was stirred at room temperature for 8 h and the progress of the reaction was monitored by TLC. Upon completion, the solvent was removed under reduced pressure and the residue was taken in water and extracted with ethyl acetate. Finally, the organic layer was washed with water and then dried over anhydrous sodium sulfate. The solvent was evaporated. The crude product was purified by column chromatography using chloroform:methanol (9:1) as an eluent. Single crystals were obtained from slow evaporation of its ethylacetate solution.

### S3. Refinement

The C-bound hydrogen atom were fixed geometrically (C—H = 0.93–0.97 Å) and allowed to ride on their parent atoms with *U*_iso_(H) = 1.2–1.5*U*_eq_(C). The N-bound H atom was included in the model with N—H = 0.86 Å, and with *U*_iso_(H) = 1.2*U*_eq_(N).

### Crystal data

**Table:**

C_13_H_14_N_2_O_2_	*Z* = 4
*M**_r_* = 230.26	*F*(000) = 488
Monoclinic, *P*2_1_/*n*	*D*_x_ = 1.284 Mg m^−^^3^
Hall symbol: -P 2yn	Cu *K*α radiation, λ = 1.54178 Å
*a* = 13.7183 (10) Å	µ = 0.72 mm^−^^1^
*b* = 6.2040 (5) Å	*T* = 296 K
*c* = 15.1944 (11) Å	Block, colourless
β = 112.865 (3)°	0.23 × 0.22 × 0.21 mm
*V* = 1191.56 (16) Å^3^	

### Data collection

**Table:**

Bruker X8 Proteum diffractometer	1742 reflections with *I* > 2σ(*I*)
Detector resolution: 10.7 pixels mm^-1^	*R*_int_ = 0.027
φ and ω scans	θ_max_ = 64.5°, θ_min_ = 3.7°
Absorption correction: multi-scan (*SADABS*; Sheldrick, 1997)	*h* = −15→15
*T*_min_ = 0.867, *T*_max_ = 0.867	*k* = −3→7
6730 measured reflections	*l* = −16→17
1953 independent reflections	

### Refinement

**Table:**

Refinement on *F*^2^	Secondary atom site location: difference Fourier map
Least-squares matrix: full	Hydrogen site location: inferred from neighbouring sites
*R*[*F*^2^ > 2σ(*F*^2^)] = 0.042	H-atom parameters constrained
*wR*(*F*^2^) = 0.120	*w* = 1/[σ^2^(*F*_o_^2^) + (0.0649*P*)^2^ + 0.277*P*] where *P* = (*F*_o_^2^ + 2*F*_c_^2^)/3
*S* = 1.07	(Δ/σ)_max_ = 0.001
1953 reflections	Δρ_max_ = 0.23 e Å^−^^3^
156 parameters	Δρ_min_ = −0.21 e Å^−^^3^
0 restraints	Extinction correction: *SHELXL97* (Sheldrick, 2008), FC^*^=KFC[1+0.001XFC^2^Λ^3^/SIN(2Θ)]^-1/4^
Primary atom site location: structure-invariant direct methods	Extinction coefficient: 0.0109 (13)

### Special details

**Table:**

Geometry. Bond distances, angles *etc*. have been calculated using the rounded fractional coordinates. All su's are estimated from the variances of the (full) variance-covariance matrix. The cell e.s.d.'s are taken into account in the estimation of distances, angles and torsion angles
Refinement. Refinement on *F*^2^ for ALL reflections except those flagged by the user for potential systematic errors. Weighted *R*-factors *wR* and all goodnesses of fit *S* are based on *F*^2^, conventional *R*-factors *R* are based on *F*, with *F* set to zero for negative *F*^2^. The observed criterion of *F*^2^ > σ(*F*^2^) is used only for calculating -*R*-factor-obs *etc*. and is not relevant to the choice of reflections for refinement. *R*-factors based on *F*^2^ are statistically about twice as large as those based on *F*, and *R*-factors based on ALL data will be even larger.

### Fractional atomic coordinates and isotropic or equivalent isotropic displacement parameters (Å^2^)

**Table:**

	*x*	*y*	*z*	*U*_iso_*/*U*_eq_	
O6	0.76182 (10)	−0.0363 (2)	0.94368 (9)	0.0601 (5)	
O7	0.55113 (10)	0.2685 (2)	1.07873 (8)	0.0578 (4)	
N1	0.66453 (10)	0.0760 (2)	1.02847 (9)	0.0424 (4)	
N3	0.57906 (12)	0.3540 (2)	0.94258 (10)	0.0519 (5)	
C2	0.59249 (12)	0.2404 (3)	1.02119 (11)	0.0434 (5)	
C4	0.64286 (12)	0.2741 (2)	0.89160 (11)	0.0409 (5)	
C5	0.69854 (12)	0.0850 (2)	0.95511 (11)	0.0414 (5)	
C8	0.70154 (13)	−0.0797 (3)	1.10674 (12)	0.0510 (5)	
C9	0.79910 (18)	−0.0056 (4)	1.18691 (15)	0.0760 (8)	
C10	0.57793 (11)	0.2115 (2)	0.78900 (10)	0.0383 (4)	
C11	0.50778 (13)	0.0411 (3)	0.75563 (14)	0.0545 (6)	
C12	0.45848 (16)	0.0121 (4)	0.65796 (16)	0.0713 (7)	
C13	0.47830 (15)	0.1510 (4)	0.59623 (14)	0.0741 (8)	
C14	0.54676 (14)	0.3205 (4)	0.62923 (13)	0.0649 (7)	
C15	0.59759 (11)	0.3507 (3)	0.72680 (11)	0.0447 (5)	
C16	0.67693 (15)	0.5191 (3)	0.77928 (14)	0.0586 (6)	
C17	0.72378 (15)	0.4361 (3)	0.88099 (13)	0.0569 (6)	
H1	0.85420	0.01810	1.16380	0.1140*	
H2	0.44450	0.12930	0.53070	0.0890*	
H3	0.78460	0.12630	1.21260	0.1140*	
H4	0.53720	0.46290	0.92390	0.0620*	
H5	0.55900	0.41400	0.58670	0.0780*	
H6	0.41160	−0.10190	0.63380	0.0860*	
H7	0.49420	−0.05110	0.79780	0.0650*	
H8	0.73090	0.53320	0.75320	0.0700*	
H9	0.64310	0.65790	0.77600	0.0700*	
H10	0.73560	0.55430	0.92580	0.0680*	
H11	0.79080	0.36510	0.89350	0.0680*	
H12	0.64620	−0.10350	1.13040	0.0610*	
H13	0.71580	−0.21600	1.08280	0.0610*	
H14	0.82130	−0.11370	1.23600	0.1140*	

### Atomic displacement parameters (Å^2^)

**Table:**

	*U*^11^	*U*^22^	*U*^33^	*U*^12^	*U*^13^	*U*^23^
O6	0.0622 (8)	0.0624 (8)	0.0613 (8)	0.0248 (6)	0.0301 (6)	0.0006 (6)
O7	0.0702 (8)	0.0651 (8)	0.0502 (7)	0.0260 (6)	0.0366 (6)	0.0125 (6)
N1	0.0483 (7)	0.0410 (7)	0.0397 (7)	0.0137 (5)	0.0190 (6)	0.0071 (5)
N3	0.0724 (9)	0.0482 (8)	0.0425 (8)	0.0301 (7)	0.0305 (7)	0.0115 (6)
C2	0.0505 (9)	0.0436 (8)	0.0382 (8)	0.0131 (7)	0.0195 (7)	0.0021 (7)
C4	0.0489 (8)	0.0398 (8)	0.0373 (8)	0.0060 (6)	0.0205 (7)	−0.0005 (6)
C5	0.0428 (8)	0.0411 (8)	0.0400 (8)	0.0059 (7)	0.0159 (6)	−0.0035 (6)
C8	0.0549 (9)	0.0473 (9)	0.0523 (10)	0.0120 (7)	0.0225 (8)	0.0144 (8)
C9	0.0771 (13)	0.0799 (15)	0.0547 (12)	0.0073 (11)	0.0079 (10)	0.0142 (10)
C10	0.0360 (7)	0.0432 (8)	0.0382 (8)	0.0043 (6)	0.0171 (6)	0.0003 (6)
C11	0.0482 (9)	0.0568 (10)	0.0589 (11)	−0.0082 (8)	0.0211 (8)	−0.0026 (8)
C12	0.0516 (10)	0.0831 (14)	0.0681 (14)	−0.0145 (10)	0.0112 (10)	−0.0207 (11)
C13	0.0497 (10)	0.1212 (19)	0.0424 (10)	−0.0016 (12)	0.0080 (8)	−0.0116 (11)
C14	0.0503 (10)	0.1028 (16)	0.0408 (10)	0.0043 (10)	0.0167 (8)	0.0148 (10)
C15	0.0367 (8)	0.0571 (10)	0.0420 (9)	0.0049 (7)	0.0171 (7)	0.0084 (7)
C16	0.0587 (10)	0.0530 (10)	0.0633 (12)	−0.0060 (8)	0.0230 (9)	0.0116 (9)
C17	0.0621 (10)	0.0521 (10)	0.0516 (10)	−0.0131 (8)	0.0167 (8)	−0.0065 (8)

### Geometric parameters (Å, º)

**Table:**

O6—C5	1.211 (2)	C14—C15	1.384 (2)
O7—C2	1.226 (2)	C15—C16	1.496 (3)
N1—C2	1.394 (2)	C16—C17	1.515 (3)
N1—C5	1.367 (2)	C8—H12	0.9700
N1—C8	1.462 (2)	C8—H13	0.9700
N3—C2	1.337 (2)	C9—H1	0.9600
N3—C4	1.462 (2)	C9—H3	0.9600
N3—H4	0.8600	C9—H14	0.9600
C4—C5	1.523 (2)	C11—H7	0.9300
C4—C10	1.515 (2)	C12—H6	0.9300
C4—C17	1.552 (3)	C13—H2	0.9300
C8—C9	1.490 (3)	C14—H5	0.9300
C10—C11	1.386 (2)	C16—H8	0.9700
C10—C15	1.382 (2)	C16—H9	0.9700
C11—C12	1.383 (3)	C17—H10	0.9700
C12—C13	1.376 (3)	C17—H11	0.9700
C13—C14	1.369 (3)		
			
O6···C17^i^	3.392 (2)	C17···H2^x^	3.0300
O6···C14^ii^	3.343 (3)	H1···C5	3.0900
O7···N3^iii^	2.886 (2)	H1···C13^x^	3.0900
O6···H11	2.6800	H1···C14^x^	3.0600
O6···H13	2.6700	H2···C13^viii^	3.0700
O6···H5^ii^	2.6900	H2···C17^ix^	3.0300
O6···H10^i^	2.5600	H2···H11^ix^	2.3200
O7···H12	2.6200	H3···H14^iv^	2.4900
O7···H14^iv^	2.7700	H4···O7^iii^	2.0500
O7···H4^iii^	2.0500	H4···C2^iii^	2.9000
O7···H7^v^	2.5800	H5···O6^vi^	2.6900
N3···O7^iii^	2.886 (2)	H7···C5	3.0100
C14···O6^vi^	3.343 (3)	H7···O7^v^	2.5800
C17···O6^vii^	3.392 (2)	H8···C15^vi^	2.9900
C2···H4^iii^	2.9000	H9···C11^vii^	2.9600
C5···H1	3.0900	H10···O6^vii^	2.5600
C5···H7	3.0100	H11···O6	2.6800
C11···H9^i^	2.9600	H11···H2^x^	2.3200
C13···H2^viii^	3.0700	H12···O7	2.6200
C13···H1^ix^	3.0900	H13···O6	2.6700
C14···H1^ix^	3.0600	H14···O7^xi^	2.7700
C15···H8^ii^	2.9900	H14···H3^xi^	2.4900
			
C2—N1—C5	111.19 (12)	C4—C17—C16	106.81 (15)
C2—N1—C8	124.02 (14)	N1—C8—H12	109.00
C5—N1—C8	124.75 (14)	N1—C8—H13	109.00
C2—N3—C4	113.05 (14)	C9—C8—H12	109.00
C2—N3—H4	123.00	C9—C8—H13	109.00
C4—N3—H4	123.00	H12—C8—H13	108.00
N1—C2—N3	107.70 (14)	C8—C9—H1	109.00
O7—C2—N1	123.90 (15)	C8—C9—H3	109.00
O7—C2—N3	128.41 (17)	C8—C9—H14	109.00
N3—C4—C17	115.79 (12)	H1—C9—H3	110.00
C5—C4—C10	113.84 (11)	H1—C9—H14	109.00
C5—C4—C17	111.22 (14)	H3—C9—H14	109.00
C10—C4—C17	102.56 (13)	C10—C11—H7	121.00
N3—C4—C5	100.46 (12)	C12—C11—H7	121.00
N3—C4—C10	113.47 (14)	C11—C12—H6	120.00
O6—C5—N1	125.62 (14)	C13—C12—H6	120.00
N1—C5—C4	107.59 (13)	C12—C13—H2	119.00
O6—C5—C4	126.79 (15)	C14—C13—H2	119.00
N1—C8—C9	112.28 (16)	C13—C14—H5	121.00
C4—C10—C15	110.52 (13)	C15—C14—H5	120.00
C4—C10—C11	128.23 (14)	C15—C16—H8	111.00
C11—C10—C15	121.24 (15)	C15—C16—H9	111.00
C10—C11—C12	118.18 (18)	C17—C16—H8	111.00
C11—C12—C13	120.4 (2)	C17—C16—H9	111.00
C12—C13—C14	121.37 (19)	H8—C16—H9	109.00
C13—C14—C15	119.00 (19)	C4—C17—H10	110.00
C10—C15—C16	111.52 (14)	C4—C17—H11	110.00
C10—C15—C14	119.80 (17)	C16—C17—H10	110.00
C14—C15—C16	128.68 (17)	C16—C17—H11	110.00
C15—C16—C17	103.82 (15)	H10—C17—H11	109.00
			
C5—N1—C2—O7	−179.31 (16)	N3—C4—C10—C15	112.36 (15)
C5—N1—C2—N3	0.70 (19)	C5—C4—C10—C11	45.9 (2)
C8—N1—C2—O7	−1.6 (3)	C5—C4—C10—C15	−133.54 (15)
C8—N1—C2—N3	178.45 (15)	C17—C4—C10—C11	166.16 (17)
C2—N1—C5—O6	179.35 (16)	C17—C4—C10—C15	−13.28 (17)
C2—N1—C5—C4	−0.48 (17)	N3—C4—C17—C16	−103.10 (17)
C8—N1—C5—O6	1.6 (3)	C5—C4—C17—C16	143.07 (15)
C8—N1—C5—C4	−178.21 (14)	C10—C4—C17—C16	21.02 (17)
C2—N1—C8—C9	−90.8 (2)	C4—C10—C11—C12	−178.69 (18)
C5—N1—C8—C9	86.6 (2)	C15—C10—C11—C12	0.7 (3)
C4—N3—C2—O7	179.36 (17)	C4—C10—C15—C14	179.40 (16)
C4—N3—C2—N1	−0.64 (19)	C4—C10—C15—C16	0.3 (2)
C2—N3—C4—C5	0.34 (17)	C11—C10—C15—C14	−0.1 (3)
C2—N3—C4—C10	122.24 (15)	C11—C10—C15—C16	−179.18 (16)
C2—N3—C4—C17	−119.53 (16)	C10—C11—C12—C13	−0.7 (3)
N3—C4—C5—O6	−179.74 (16)	C11—C12—C13—C14	0.0 (4)
N3—C4—C5—N1	0.09 (16)	C12—C13—C14—C15	0.6 (3)
C10—C4—C5—O6	58.6 (2)	C13—C14—C15—C10	−0.6 (3)
C10—C4—C5—N1	−121.54 (15)	C13—C14—C15—C16	178.3 (2)
C17—C4—C5—O6	−56.6 (2)	C10—C15—C16—C17	13.2 (2)
C17—C4—C5—N1	123.20 (14)	C14—C15—C16—C17	−165.8 (2)
N3—C4—C10—C11	−68.2 (2)	C15—C16—C17—C4	−21.00 (19)

Symmetry codes: (i) *x*, *y*−1, *z*; (ii) −*x*+3/2, *y*−1/2, −*z*+3/2; (iii) −*x*+1, −*y*+1, −*z*+2; (iv) −*x*+3/2, *y*+1/2, −*z*+5/2; (v) −*x*+1, −*y*, −*z*+2; (vi) −*x*+3/2, *y*+1/2, −*z*+3/2; (vii) *x*, *y*+1, *z*; (viii) −*x*+1, −*y*, −*z*+1; (ix) *x*−1/2, −*y*+1/2, *z*−1/2; (x) *x*+1/2, −*y*+1/2, *z*+1/2; (xi) −*x*+3/2, *y*−1/2, −*z*+5/2.

### Hydrogen-bond geometry (Å, º)

**Table:**

*D*—H···*A*	*D*—H	H···*A*	*D*···*A*	*D*—H···*A*
N3—H4···O7^iii^	0.86	2.05	2.886 (2)	163
C11—H7···O7^v^	0.93	2.58	3.501 (2)	173
C17—H10···O6^vii^	0.97	2.56	3.392 (2)	143

Symmetry codes: (iii) −*x*+1, −*y*+1, −*z*+2; (v) −*x*+1, −*y*, −*z*+2; (vii) *x*, *y*+1, *z*.
