# Supplementary figures and images for: Crystal structure of 1-ethyl­spiro­[imid­az­olidine-4,1′-indane]-2,5-dione
Source: Acta Crystallogr Sect E Struct Rep Online. 2014 Aug 1;70(Pt 9):o954. doi: 10.1107/S1600536814017097 (PMC4186094; doi:10.1107/S1600536814017097)

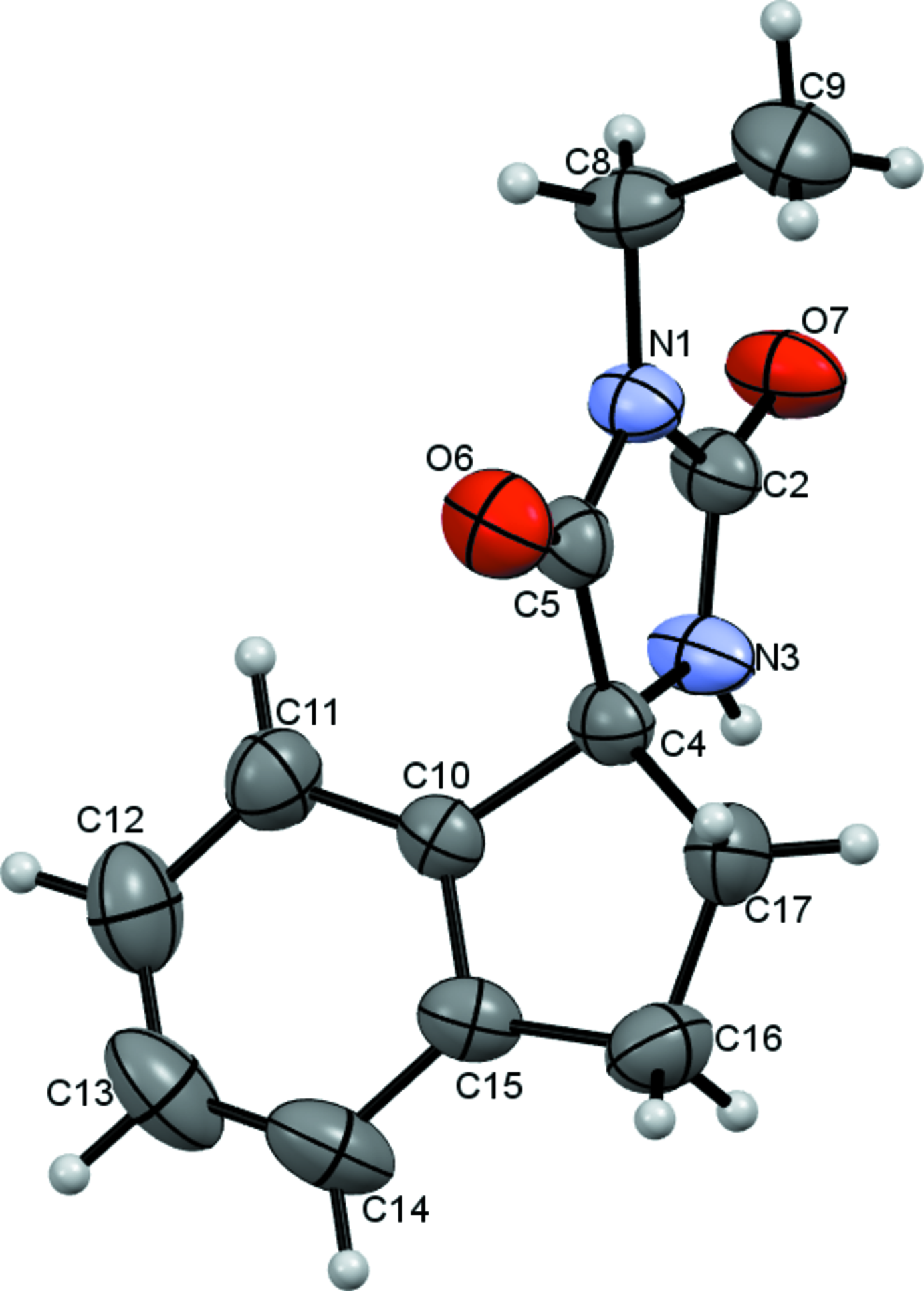

Supplement: Supplementary file 4 [file e-70-0o954-fig1.tif]

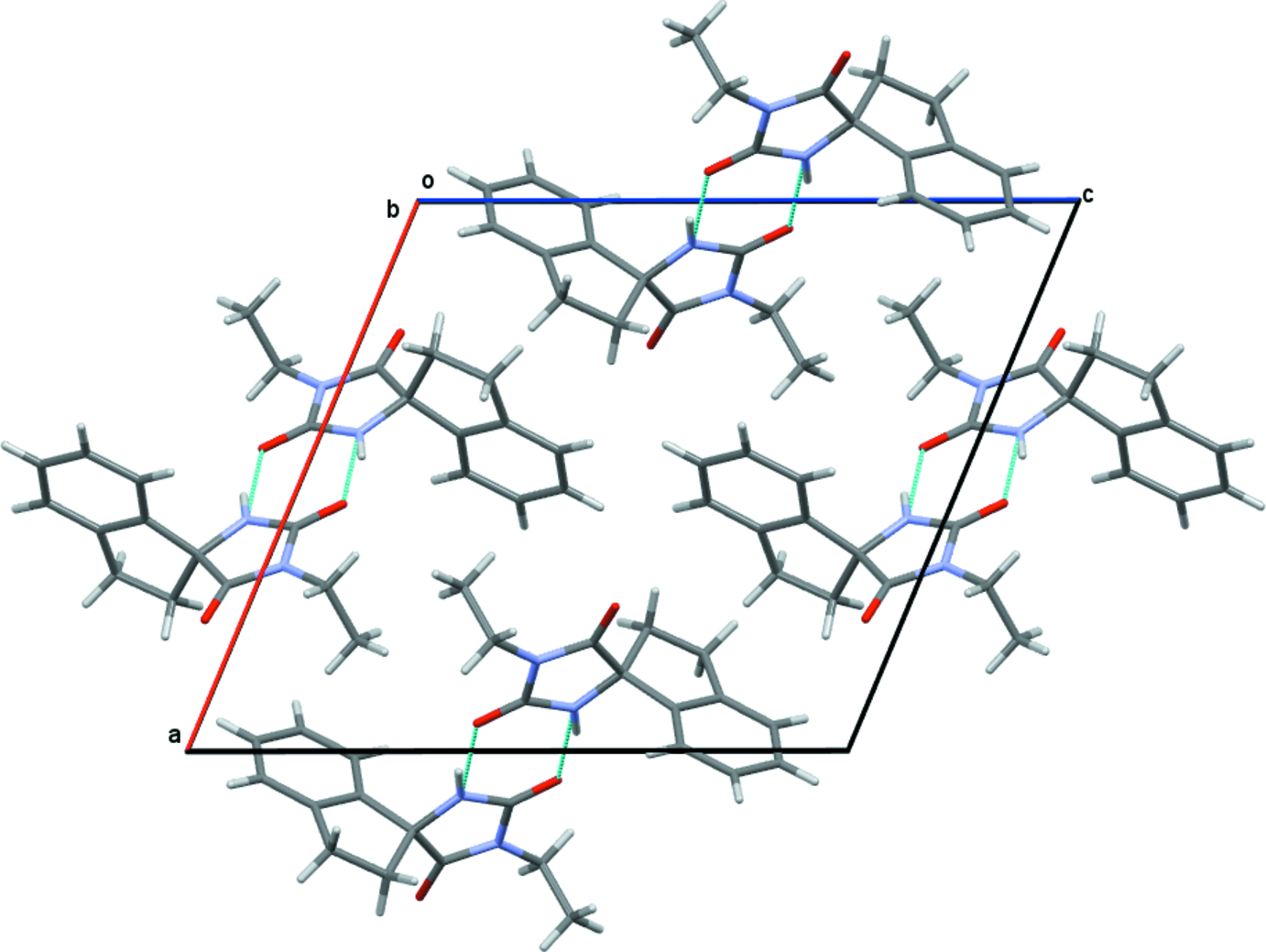

Supplement: Supplementary file 5 [file e-70-0o954-fig2.tif]
